# Outcomes of Cataract Surgery in Diabetic Patients in King Abdulaziz Medical City in 2019

**DOI:** 10.7759/cureus.30216

**Published:** 2022-10-12

**Authors:** Dana K Alsarhani, Ghida S Altammami, Hadeel T Alzahrani, Rawan M Alhazmi, Shuq A Alanazi, Shiji Gangadhanan, Abdulaziz Alhowass

**Affiliations:** 1 Ophthalmology, King Saud Bin Abdulaziz University for Health Sciences College of Medicine, Riyadh, SAU; 2 Ophthalmology, King Abdulaziz Medical City Riyadh, Riyadh, SAU

**Keywords:** macular edema, comorbidites, postoperative complications, cataract, diabetes

## Abstract

Background

In this study, we aimed to analyze various complications following cataract surgery in diabetic patients and compare the outcomes of diabetic patients with comorbidities versus diabetic patients without comorbidities.

Methodology

This study was conducted in the adult ophthalmology department at a tertiary teaching care center. A retrospective cross-sectional chart review was conducted from January 1, 2019, to December 31, 2019. The selection was made using a non-probability consecutive sampling technique with a data collection sheet to include all male and female Saudi diabetic patients 40-80 years old who underwent cataract surgery in 2019. The data were divided into diabetic patients with comorbidities and diabetic patients without comorbidities to assess the postoperative complications in both groups. SPSS version 26 (IBM Corp., Armonk, NY, USA) was used for data analysis.

Results

This study analyzed 290 diabetic patients; the most common age group was more than 65 years old (150, 51.7%), with slightly more females (147, 50.7%). A total of 181 (62.7%) patients had complications after surgery, and 255 (87.9%) patients had comorbidities. The most reported complication was corneal edema (181, 62.4%). Additionally, hypertension was the most frequently reported comorbidity (206, 71%). We also found that complications after cataract surgery were more common among females (p = 0.025).

Conclusions

The most prevalent postoperative cataract surgery complication was corneal edema in 181 (62.4%) patients. Despite comorbidities, no changes were reported in the prevalence of postoperative complications.

## Introduction

An International Diabetes Federation study estimated that the prevalence of diabetes mellitus (DM) will be 439 million patients by 2030 [1]. Another study ranked Saudi Arabia seventh worldwide and second in the Middle East for the prevalence rate of diabetes [2]. One of the complications of diabetes is diabetic retinopathy, the most common microvascular dysfunction affecting the eye [3,4]. Other ocular tissues, such as the cornea, tear film, and crystalline lens, are also affected early in diabetic patients, which may lead to visual dysfunction [5,6]. The remarkable association between cataract development and diabetes was highlighted by a study called the Beaver Dam Eye, which reported that an increased incidence of posterior and subcapsular cataract progression correlates with diabetes [7]. Further, the Framingham study showed that 65-year-old diabetic patients have a four-fold increased risk of developing cataracts, which further increases two-fold in above 65-year-old diabetic patients [7]. Evidence revealed that the intraocular level of inflammatory cytokines and vascular endothelial growth factor (VEGF) can be further increased by cataract surgery with diabetic retinopathy [8]. Such cytokines may clinically or subclinically worsen diabetic retinopathy and maculopathy [9]. Recent studies have reported some postoperative complications relating to cataract surgery commonly found in diabetic patients, including posterior capsular opacification, corneal edema, macular edema, and astigmatism [10-13].

Although many studies provide clear evidence of cataract formation and progression in diabetic patients, significant ambiguity about the correlation between diabetes and postoperative cataract surgery complications holds an immense burden. Additionally, there is a prodigious lack of research, especially in Saudi Arabia, specifically on whether the presence of other comorbidities could be a defining factor.

This study was designed to assess the hypothesis that cataract surgery in diabetic patients is associated with an increased risk of postoperative complications. To investigate the complexity of the disease, nature, and course of diabetes, as well as its effect on the eyes, especially in the context of cataract surgery, we collected retrospective data of patients who underwent cataract surgery along with their visual outcomes. Furthermore, we aimed to fill the gap in this subject as no previous studies have been conducted on the topic, and a better understanding of various complications of cataract surgery leading to poor outcomes in diabetic patients may guide better overall management of these patients.

## Materials and methods

A retrospective, cross-sectional, chart review of all diabetic patients who underwent cataract surgery from January 1, 2019, until December 31, 2019, was conducted using the electronic medical records of a teaching tertiary care center (1,501-bed capacity). The Raosoft sample size calculator was used to calculate the sample size, with a margin of error of 5%, a confidence level of 95%, and a response distribution of 50%. The required sample size was 290, and the population was 1,191. The non-probability consecutive sampling technique was used to review and collect patient files for data collection in the study. Therefore, the selection was made using a data collection form to select Saudi diabetic patients between 40 and 80 years old who underwent cataract surgery in 2019, of both genders, with and without comorbidities.

Statistical analyses were carried out using SPSS version 26 (IBM Corp., Armonk, NY, USA). Continuous variables were expressed as mean and standard deviation (SD), whereas categorical variables were expressed as frequencies (%). The chi-square test was used to compare diabetic patients with comorbidities who underwent cataract surgery and diabetic patients without comorbidities who underwent cataract surgery. Descriptive statistics were presented using numbers and percentages (%). The relationship between the complications and comorbidities among the demographic characteristics of diabetic patients was assessed using the chi-square test. A p-value of <0.05 (two-sided) was used to indicate statistical significance. Patient privacy and confidentiality were assured, names/ID numbers were not collected, and a serial code was given to each data collection sheet. All data were maintained in a secure, password-protected computer.

## Results

This study analyzed the data of 290 diabetic patients. Table 1 describes the basic demographic characteristics of the patients. The most common age group was more than 65 years (150, 51.7%), with slightly more females (147, 50.7%). The prevalence of patients who had complications after surgery was 181 (62.7%), while the prevalence of patients with associated comorbidities was 225 (87.9%) (Table 1).

**Table 1:** Demographics and complications of diabetic patients after cataract surgery (n = 290).

Study variables	N (%)
Age group	
≤65 years	140 (48.3%)
>65 years	150 (51.7%)
Gender	
Male	143 (49.3%)
Female	147 (50.7%)
Complications after surgery	
No	109 (37.6%)
Yes	181 (62.7%)
Comorbidities	
No	35 (12.1%)
Yes	255 (87.9%)

**Figure 1:**
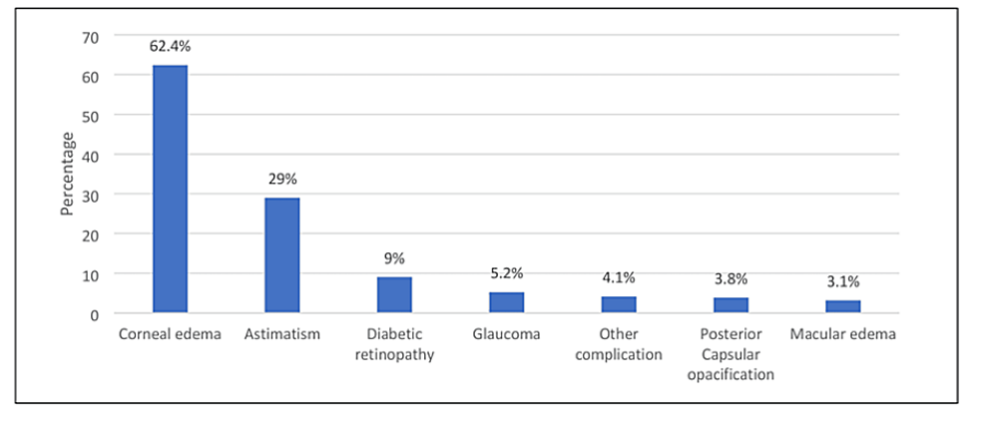
Complications of diabetic patients after cataract surgery.

Figure 1 presents the complications of diabetic patients after surgery. The most reported complication was corneal edema (181, 62.4%), followed by astigmatism (84, 29%), diabetic retinopathy (26, 9%), and macular edema (9, 3.1%).

**Figure 2:**
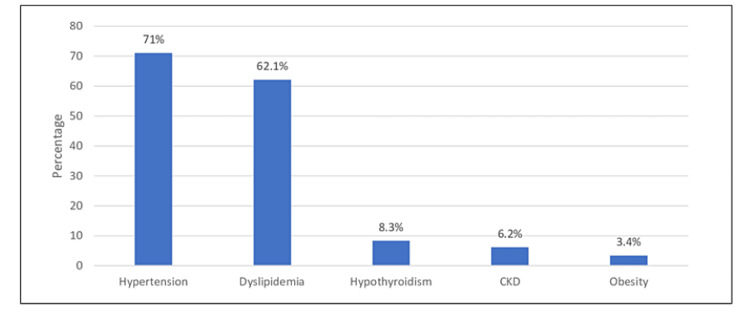
Associated comorbidities of diabetic patients.

Figure 2 shows the associated comorbidities of diabetic patients who underwent cataract surgery. Hypertension was the most frequently reported comorbidity (206, 71%), followed by dyslipidemia (181, 62.1%), hypothyroidism (24, 8.3%), and obesity (10, 3.4%).

When measuring the relationship between the complications of surgery concerning the demographic characteristics of patients who underwent cataract surgery, we observed that complications after surgery were more common among females (p = 0.025), while complications in age group, comorbidities, and specific comorbidities were not significantly different (p > 0.05) (Table 2).

**Table 2:** Relationship between complications after surgery and the demographic characteristics of patients who underwent cataract surgery (n = 290). *: variables with multiple response answers; §: p-value calculated using the chi-square test; **: significant at p < 0.05 level.

Factor	Complications after surgery		P-value^§^
	No, (%) (n = 109)	Yes, N (%) (n = 181)	
Age group			
≤65 years	52 (47.7%)	88 (48.6%)	0.880
>65 years	57 (52.3%)	93 (51.4%)	
Gender			
Male	63 (57.8%)	80 (44.2%)	0.025**
Female	46 (42.2%)	101 (55.8%)	
Comorbidities			
No	12 (11.0%)	23 (12.7%)	0.667
Yes	97 (89.0%)	158 (87.3%)	
Specific comorbidities*			
Hypertension	80 (73.4%)	126 (69.6%)	0.492
Dyslipidemia	68 (37.6%)	69 (38.1%)	0.931
Hypothyroidism	09 (08.3%)	15 (08.3%)	0.993
Chronic kidney disease	09 (08.3%)	09 (05.0%)	0.262
Obesity	06 (05.5%)	04 (02.2%)	0.136

When measuring the relationships between comorbidities, complications, and demographic characteristics, we found that the prevalence of patients with comorbidities was significantly higher in males (p = 0.015), while comorbidities in the age group and the specific complication of patients after surgery were not significantly different across groups (p > 0.05) (Table 3).

**Table 3:** Relationship between having comorbidities among the demographic characteristics and complications of patients who underwent cataract surgery (n = 290). *: variables with multiple response answers; ^§^: p-value calculated using the chi-square test; **: significant at p < 0.05 level.

Factor	Comorbidities		P-value^§^
	No, N (%) (n = 35)	Yes, N (%) (n = 255)	
Age group			
≤65 years	21 (60.0%)	119 (46.7%)	0.139
>65 years	14 (40.0%)	136 (53.3%)	
Gender			
Male	24 (68.6%)	119 (46.7%)	0.015**
Female	11 (31.4%)	136 (53.3%)	
Complications of patients*			
Posterior capsular opacification	2 (05.7%)	9 (03.5%)	0.526
Corneal edema	12 (34.3%)	97 (38.0%)	0.667
Macular edema	0	9 (03.5%)	0.259
Astigmatism	13 (37.1%)	71 (27.8%)	0.255
Diabetic retinopathy	6 (17.1%)	20 (07.1%)	0.071
Glaucoma	2 (05.7%)	13 (05.1%)	0.877
Other complications	1 (02.9%)	11 (04.3%)	0.685

Analytical analysis

Factors of Complications After Surgery

Female diabetic patients were more associated with complications after cataract surgery (χ^2^ = 5.034; p = 0.025). However, age group (χ^2^ = 0.023l; p = 0.880), comorbidities (χ^2^ = 0.185; p = 0.667), hypertension (χ^2^ = 0.473; p = 0.492), dyslipidemia (χ^2^ = 0.007; p = 0.931), hypothyroidism (χ^2^ = 0.000l p = 0.993), chronic kidney disease (χ^2^ = 1.261; p = 0.262), and obesity (χ^2^ = 2.218; p = 0.136) were not significant factors of complications after cataract surgery.

Factors of Comorbidities

Male diabetic patients were significantly more likely to have associated comorbidities (χ^2^ = 5.908; p = 0.015), while age group (χ^2^ = 2.191; p = 0.139) and complications of surgery [posterior capsule opacification (χ^2^ = 0.403; p = 0.526), corneal edema (χ^2^ = 0.185; p = 0.667), macular edema (χ^2^ = 1.275; p = 0.259), astigmatism (χ^2^ = 1.294; p = 0.255), diabetic retinopathy (χ^2^ = 3.261; p = 0.071), glaucoma (χ^2^ = 0.024; p = 0.877), and other complications (χ^2^ = 0.165; p = 0.685)] had no significant relationships with comorbidities.

## Discussion

This study was conducted to identify the outcomes of cataract surgery among diabetic patients. From an initial 1,191 patients in a tertiary care center who underwent cataract surgery in 2019, after applying the exclusion criteria, 290 Saudi males and females aged between 40 and 80 years were enrolled in the study. Corneal edema was the most common postoperative complication in diabetic eyes after cataract surgery. This finding is similar to the corneal edema study after phacoemulsification surgery in patients with type II DM, as it was found that postoperative corneal edema was significantly higher in diabetic patients [14]. Moreover, the established proportion of new-onset and progressive postoperative diabetic retinopathy was almost significant (p = 0.07). Likewise, a previous study concluded that operated diabetic eyes had a higher rate of diabetic retinopathy progression, but this was not significant [15]. Furthermore, a significant relationship between cataract surgery and postoperative development of macular edema could not be determined, as reported by Guliani et al. They concluded that there was no significant difference in the mean central macular thickness values between the two groups on any of the three occasions when the central macular thickness was measured (p = 0.374 and p = 0.313 at weeks one and six, respectively) [16]. This finding was further supported by a study reporting no association between developing macular edema and cataract surgery [15].

In addition, this study aimed to compare the visual outcomes of diabetic patients who underwent cataract surgery with other comorbidities, including hypertension, obesity, hyperlipidemia, thyroid disorders, and chronic kidney disease, and diabetic patients who underwent cataract surgery without comorbidities. We found a non-significant relationship between comorbidities and postoperative complications of cataract surgery. Consistent with the study reported by Mylona et al., which stated that hypertension was the most frequent risk factor, ranging from 43.8% in patients with subcapsular cataracts, 24.3% in patients with nuclear cataracts, 28.6% in patients with cortical cataracts, and 27.6% in patients with mixed type cataracts, we observed that hypertension was the most frequently reported comorbidity in 206 (71%) patients [17]. The second most common was dyslipidemia (181, 62.1%), which was evidenced to be a contributing factor in the development of postoperative complications [18].

This study has some limitations. Diabetic patients with other comorbidities were more in number compared to diabetic patients without comorbidities, which may have led to biased representation in our results. Finally, our population included all diabetic patients who underwent cataract surgery without considering if diabetes was controlled or not, which may have affected the overall outcomes.

## Conclusions

The most reported complication was corneal edema, followed by astigmatism, diabetic retinopathy, and macular edema. Comorbidities included hypertension, dyslipidemia, hypothyroidism, and obesity. We conclude that despite the presence of comorbidities, no changes were reported in the prevalence of postoperative complications. Further studies should be conducted regarding the development of postoperative cataract surgery complications to identify patients with high-risk factors and take the necessary steps to minimize those complications.
